# An Incidental Discovery Following Hypertension and Headaches: A Pheochromocytoma Case Report

**DOI:** 10.7759/cureus.13783

**Published:** 2021-03-09

**Authors:** Geoffrey Lindblad, Stephanie Prater, Ashley Hall, Sheyla Gonzalez, Danay Herrera

**Affiliations:** 1 Radiology, Aventura Hospital and Medical Center, Aventura, USA; 2 Internal Medicine, Aventura Hospital and Medical Center, Aventura, USA

**Keywords:** pheochromocytoma, diagnostic radiology, internal medicine, endocrinology

## Abstract

Pheochromocytomas (PCC) are rare neuroendocrine tumors of the adrenal medulla that arise from chromaffin cells. These cells are neural crest derivatives and are innervated by the splanchnic nerve of the sympathetic nervous system which releases acetylcholine that in turn binds to nicotinic acetylcholine receptors of the adrenal medulla causing the release of catecholamines. The dopamine, norepinephrine, and epinephrine released from these tumors are responsible for the episodic hyperadrenergic symptoms seen in these cases such as hypertension, palpitations, and headaches.

This case report discusses the incidental finding of a unilateral PCC in a 58-year-old woman who initially presented to our emergency department complaining of intermittent chest pain and headaches for two days.

## Introduction

Pheochromocytomas (PCC) are chromaffin cell tumors that arise from the adrenal medulla; however, chromaffin cell tumors can also be found outside of the adrenal gland in which they are designated as paragangliomas (PGL). PGL tumors are further classified in origin as sympathetic or parasympathetic. The sympathetic PGL arise from the sympathetic plexus and ganglia of the thorax, abdomen, and pelvis. In contrast, the parasympathetic PGL are located in the head and neck [1].

It is estimated that roughly 6% of patients with reported incidental adrenal masses are ultimately diagnosed with a PCC [2,3]. An epidemiological pathology study in the Netherlands performed over the years of 1995 to 2015 found the yearly incidence rate of PCC or PGL to be approximately 0.57 cases per 100,000 persons, illustrating how rarely these tumors are clinically encountered [4]. 

Traditionally, PCC and PGL have been reported to follow the “rule of 10s”, in that 10% are malignant, 10% occur bilaterally, 10% are extra-adrenal in origin, 10% occur in children, and 10% are familial [5]. PCC can develop in diseases associated with germline mutations in tumor suppressor genes (TSG) as well as oncogenes. Examples include but are not limited to, TSG such as neurofibromin (Ras GTPase activating protein) associated with neurofibromatosis type 1, von Hippel-Lindau (VHL; hypoxia inducible factor 1a inhibitor) associated with von Hippel-Lindau disease, and additionally, receptor tyrosine kinase (RET) oncogene associated with multiple endocrine neoplasia 2A and 2B [5].

Although the clinical presentation of a patient with a PCC can vary, manifestations of these tumors usually include a combination of chest pain, hypertension, palpitations, headaches, sweating, nausea, irritability and anxiety. 

## Case presentation

Our patient is a 58-year-old female with a past medical history of hypertension, hyperlipidemia, and insulin-dependent diabetes mellitus who presented to the emergency department of our institution after complaining of intermittent, non-radiating, sub-sternal chest pain for a duration of two days with accompanying headaches. The patient reported no specific aggravating or alleviating factors of the chest pain and headaches and stated that each episode lasted approximately 15 minutes before subsiding. 

Vitals signs on arrival included a temperature of 98.4 degrees Fahrenheit, seated blood pressure of 166/88 mmHg, respiratory rate of 17 breaths per minute, and a heart rate of 81 beats per minute. Initial electrocardiogram showed T-wave inversions in leads V4-V6 but no evidence of ST-segment elevations. The first troponin level was <0.012 ng/mL (0.000-0.034). Two additional troponin levels were followed six hours apart from each other and both yielded the same result as the first. Basic labs were ordered including a complete metabolic panel (CMP) and complete cell count (CBC). The CBC and CMP were unremarkable other than a blood glucose level of 609 mg/dL (70-110). A D-dimer was ordered and found to be elevated at 665 ng/mL (0-316). 

**Figure 1:**
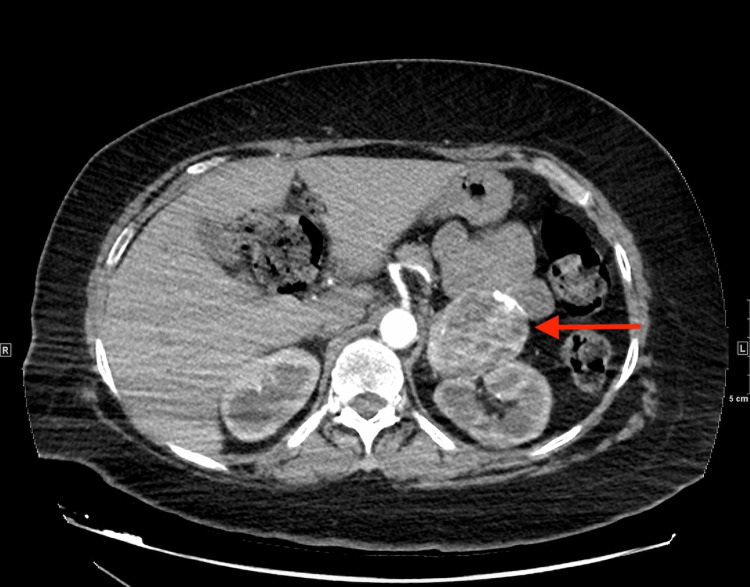
Axial view, CTA: showing a well circumscribed, left adrenal mass (red arrow) CTA: computed tomography angiography

In the setting of chest pain with an elevated D-dimer, a computed tomography angiography (CTA) of the chest was performed which was negative for pulmonary emboli and aortic dissection, however an incidental heterogeneous solid lesion adjacent to the left renal hilum was noted on the study (Figure 1).

A computed tomography (CT) scan of the abdomen and pelvis with intravenous (IV) contrast was advised by radiology after this discovery. 

**Figure 2:**
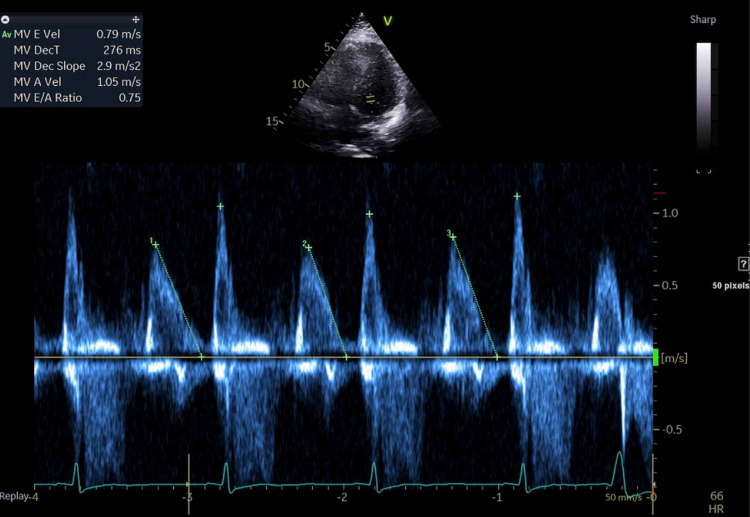
Apical short-axis view, TTE: showing a mitral valve E/A ratio of 0.75 with a deceleration time of 276 ms, consistent with a grade 1 diastolic dysfunction TTE: transthoracic echocardiogram

**Figure 3:**
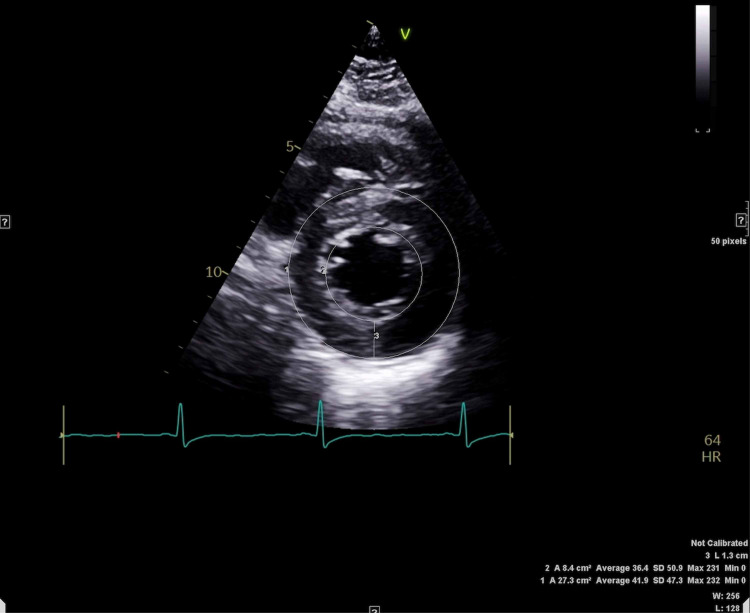
Short-axis view at level of left ventricular apex, TTE: showing left ventricle hypertrophy with concentric wall thickening up to 1.3 cm (label 3) TTE: transthoracic echocargiogram

The next day the patient underwent a pharmacological stress test and transthoracic echocardiogram that were recommended by cardiology for further evaluation of her unstable angina. No perfusion defects were evident during the stress test. The echocardiogram demonstrated an estimated visual ejection fraction of 65-75%, abnormal left ventricular relaxation with a mitral valve E/A ratio 0.75 (> 1.0) and deceleration time of 276 ms (150-240) (grade 1 diastolic dysfunction), and concentric left ventricular (LV) hypertrophy with a normal LV cavity size (Figure 2, 3). Cardiology signed off on the patient at this point. 

After cardiac etiologies of chest pain were ruled out, the CT scan of the abdomen and pelvis with IV contrast was performed which showed a heterogeneously enhancing primarily solid mass arising from the left adrenal gland, numerous simple renal cysts bilaterally, with extensive beam-hardening artifact secondary to the location of the patient's arms (images not included). 

**Figure 4:**
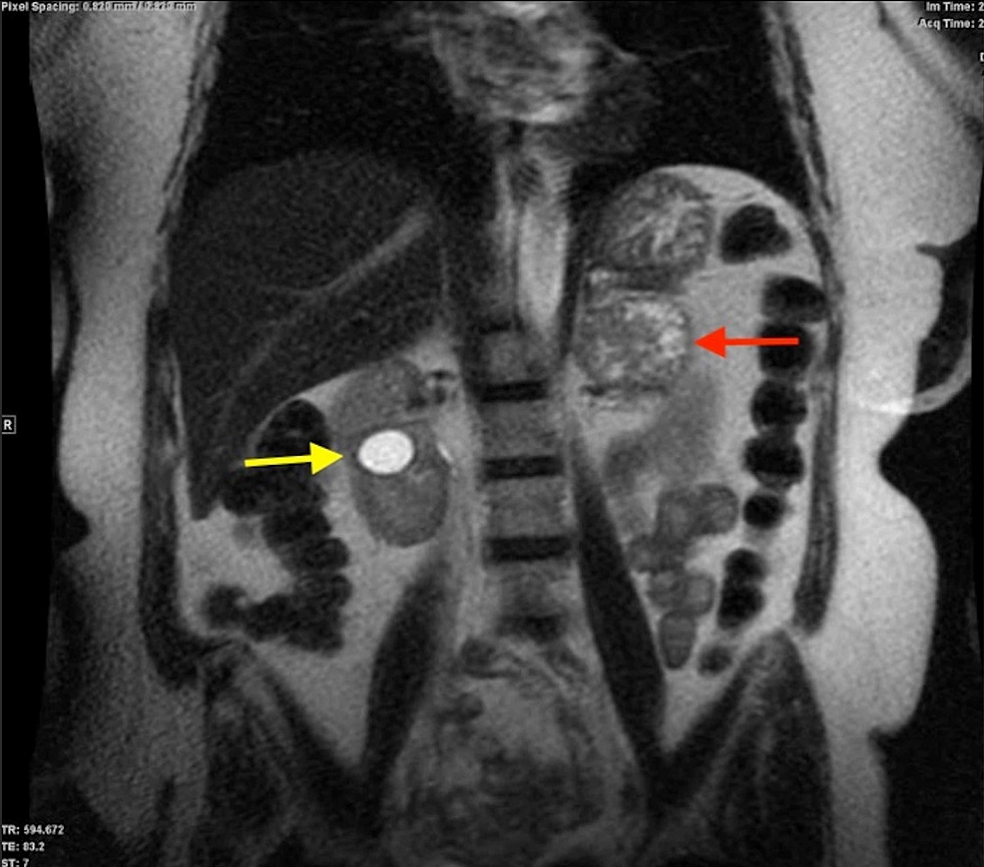
Coronal view- T2-weighted MRI showing a left adrenal mass (red arrow) and a right renal cyst (yellow arrow) MRI: magnetic resonance imaging

**Figure 5:**
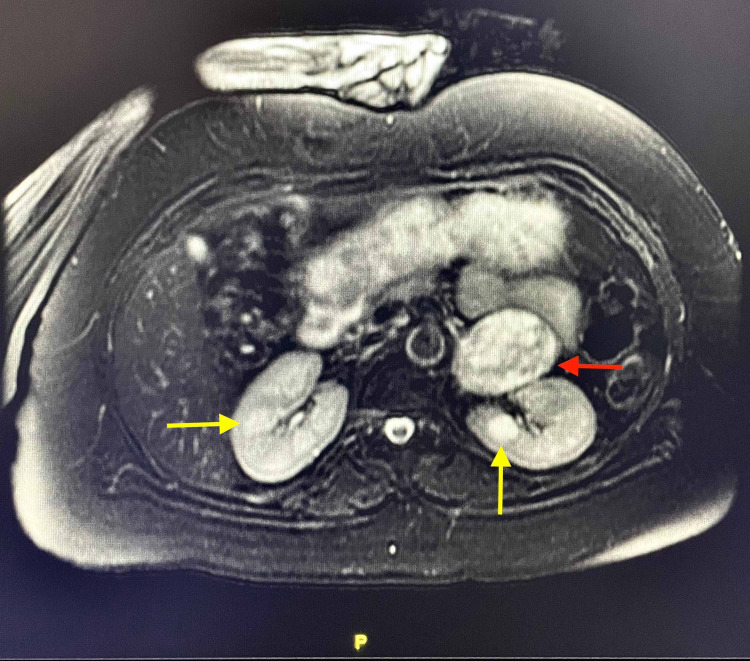
Axial view- T2-weighted MRI with fat suppression showing a left adrenal, well-circumscribed mass, measuring 4.4 x 5.7 x 5.1 cm (red arrow), and bilateral renal cysts (yellow arrows) MRI: magnetic resonance imaging

To further evaluate the left adrenal mass, a magnetic resonance imaging (MRI) of the abdomen and pelvis with and without IV contrast was ordered. The left adrenal mass was well-circumscribed, measuring 4.4 x 5.7 x 5.1 cm, with heterogeneous T2 hyperintensity favoring a pheochromocytoma (Figure 4, 5). No evidence of signal dropout on out-of-phase imaging was seen to suggest the presence of fat.

**Figure 6:**
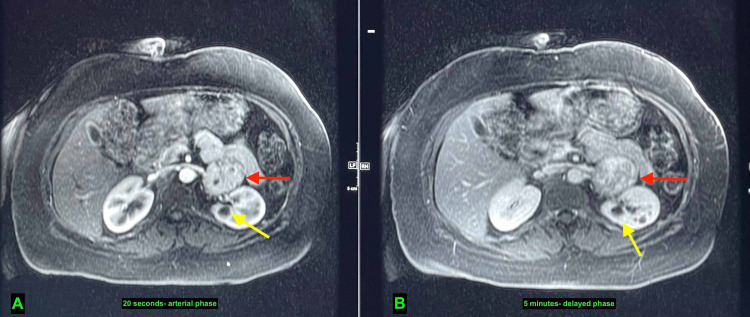
Axial view- T1-weighted post contrast MRI. A, early enhancement of left adrenal mass in arterial phase (red arrow), and renal cyst (yellow arrow). B, persistent enhancement of left adrenal mass in delayed phase (red arrow), and renal cyst (yellow arrow). MRI: magnetic resonance imaging

Persistent enhancement of the mass was evident on T1-weighted post-contrast imaging in the arterial phase (20 seconds) and throughout the delayed phase (five minutes) (Figure 6). 

**Figure 7:**
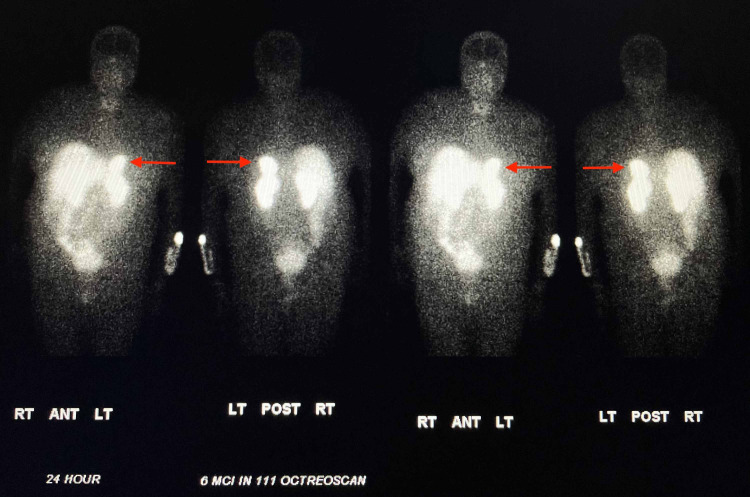
Indium-111-pentetreotide (octreoscan) showing markedly increased radiotracer uptake in the left adrenal mass (red arrows)

Our institution does not offer metaiodobenzylguanidine (MIBG) scanning, and therefore an indium-111-pentetreotide scan (octreoscan) scan was performed. The study showed markedly increased radiotracer uptake in the left adrenal mass highly suggestive of a pheochromocytoma (Figure 7). 

**Figure 8:**
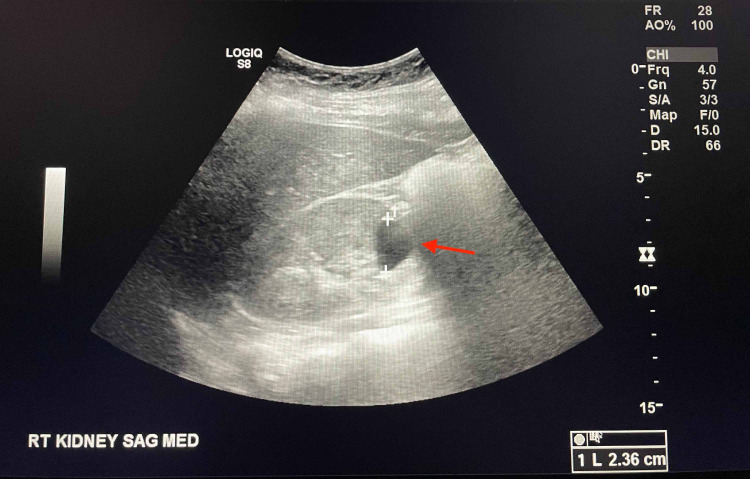
Sagittal view: ultrasonography of right kidney showing hypoechoic cystic structure (red arrow)

Furthermore, both kidneys demonstrated numerous simple cysts. A 1.6 x 1.5 x 1.8 cm complex cyst in the interpolar region of the right kidney was noted to have a small nodular area of intrinsic T1 signal favoring blood products. Subtraction images suggested a questionable enhancing solid nodular component concerning for renal cell carcinoma that was additionally evaluated with ultrasonography. On ultrasound, the complex cyst was reported to be hypoechoic and likely to be hemorrhagic in nature (Figure 8). 

Endocrinology and surgical oncology were consulted following the discovery of the adrenal mass and a more extensive endocrinology workup was completed prior to surgical planning. Plasma normetanephrine and 24-hour urine normetanephrine levels were found to be 2163.9 pg/mL (0.0-136.8) and 7450 ug/24 hour (131-612) respectively. Plasma metanephrines resulted within range at 81.9 pg/mL (0.0-88.0) while 24-hour urine metanephrines were elevated at 844 ug/24 hour (36-209).

The patient was started on oral prazosin 1mg to take twice daily for a duration of 10 days for preoperative alpha blockade prior to undergoing a left-side adrenalectomy. The surgery was completed two weeks after hospital discharge and was successful without complications and the patient made a full recovery.

## Discussion

Adrenal masses are often discovered incidentally. Differential diagnoses for unilateral adrenal masses include PCC, adrenal cortical carcinoma, adrenal metastasis, adrenal myelolipoma, adrenal adenoma, and more.

Biochemical testing and radiological studies help discern between the possible differentials and help guide physicians toward making the appropriate diagnosis.

Plasma levels and 24-hour urinary excretion of metaneprhines and normetanephrines are typically elevated in cases of PCC, as evidenced by our patient.

Initial imaging to evaluate for adrenal masses include CT and MRI studies with and without IV contrast. The sensitivity of CT scans for adrenal masses can approach 100%, although it lacks the specificity of MRI and functional imaging for PCC [6]. As illustrated in our imaging above, PCC tend to have heterogenously enhancing, increased signal on T2-weighted MRI. Functional studies such as 123 I-metaiodobenzylguanidine scan, indium-111-pentetreotide scan, and fluorodeoxyglucose positron emission tomography (FDG-PET) CT scan are commonly ordered following initial CT and MRI for further characterization of a lesion [6]. As previously mentioned, our institution does not have MIBG capabilities and therefore an octreoscan was performed instead. The results showed markedly increased radiotracer uptake which is strongly associated with PCC.

The standard of care for managing a PCC is surgical removal of the tumor. Surgical approaches can vary case by case, but typically include a transabdominal laparoscopic adrenalectomy or posteriorly, a retroperitoneoscopic adrenalectomy. According to Dickson et al., the posterior method has been shown to be a shorter operation, with decreased complications, blood loss, and hospital stay when compared to the transabdominal approach [7].

Prior to undergoing an adrenalectomy, patients with PCC are usually started on a course of alpha-blockers to prevent intraoperative hypertensive crisis. In similar fashion to the management of cocaine intoxication, sole use of beta-blockers is contraindicated in patients with PCC as unopposed alpha agonism can precipitate a hypertensive crisis. Our patient was discharged with a two-week course of prazosin for preoperative alpha blockade.

## Conclusions

The presented case report discusses a diagnostic workup for an incidental adrenal mass and the need for high clinical suspicion of a PCC when a patient presents with hypertension and headaches. Correlating the clinical presentation of our patient with the imaging findings from the CT, MRI, and octreoscan directed us to pursue further biochemical testing to confirm our suspected diagnosis at that time. Plasma and 24-hour urine metanephrines and normetanephrines were found to be elevated and the diagnosis was confirmed. The patient underwent an elective retroperitoneoscopic left adrenalectomy with no complications reported so far.
